# PPARα Antagonism Rescues Chlorpyrifos-Induced Neuro-Visual Toxicity in Zebrafish (*Danio rerio*) Larvae

**DOI:** 10.3390/toxics14030234

**Published:** 2026-03-09

**Authors:** Yuyao Jiang, Zijie Ding, Ruolin Hu, Jason T. Magnuson, Shiyan Li, Dingnan Wang, Shengli Zhou, Yirong Guo, Yang Wang, Yuanyuan Liu, Shuying Li, Wenjun Gui

**Affiliations:** 1 Institute of Pesticide and Environmental Toxicology, College of Agriculture and Biotechnology, Zhejiang University, Hangzhou 310058, China; 2 Department of Environmental Sciences, University of California, Riverside, Riverside, CA 92521, USA; 3 Zhejiang Fisheries Technical Extension Center, Hangzhou 310023, China; 4 Ecological and Environmental Monitoring Center of Zhejiang Province, Hangzhou 310012, China; 5 Zhejiang Key Laboratory of Biology and Ecological Regulation of Crop Pathogens and Insects, Ministry of Agriculture Key Laboratory of Molecular Biology of Crop Pathogens and Insect Pests, Zhejiang University, Hangzhou 310058, China

**Keywords:** chlorpyrifos, behavior, omics, peroxisome proliferator-activated receptor, toxic optic neuropathy

## Abstract

With the global population predicted to reach 10 billion by 2050, pesticides are essential for agricultural production. However, they can introduce chemical stressors into aquatic ecosystems. Chlorpyrifos (CPF) is a widely used organophosphate insecticide that can enter aquatic environments and poses potential risks to early-life-stage fish. Because the retina is an extension of the central nervous system and vision-guided behaviors are highly sensitive to neural dysfunction, we hypothesized that CPF exposure disrupts neurobehavioral and visual function via oxidative stress and PPARα-related signaling. Zebrafish larvae were exposed to CPF (0.01, 0.1, 1, 10, and 100 μg/L) with a vehicle control (VC). During the photomotor response assay, exposure to 100 μg/L CPF reduced overall swimming activity by 48.90% and dark-period activity by 57.71%, whereas 1 μg/L CPF modestly increased total distance by 6.96% (*p* = 0.003) and dark-period distance by 5.40% (*p* = 0.011). Transcriptomic profiling highlighted nervous- and vision-related functional categories, and Kyoto Encyclopedia of Genes and Genomes (KEGG) enrichment implicated pathways including gonadotropin-releasing hormone (GnRH), mitogen-activated protein kinase (MAPK), and peroxisome proliferator-activated receptor (PPAR) signaling. Targeted neurotransmitter metabolomics showed significant increases in dopamine, γ-aminobutyric acid (GABA), and acetylcholine across treatment groups, indicating broad neurotransmitter dysregulation. Consistent with these findings, neuronal fluorescence in *Tg* (*elavl3*: EGFP) larvae decreased by 12.1% and 32.5% in the 1 and 100 μg/L groups, respectively (*p* < 0.001), and glial fibrillary acidic protein (GFAP) immunofluorescence increased in the eye/brain/olfactory bulb at 1 μg/L (*p* = 0.037) and 100 μg/L (*p* = 0.002). Histology further showed retinal injury, with a 14.3% reduction in photoreceptor layer thickness at 100 μg/L (*p* = 0.034). Mechanistically, coexposure to a PPARα antagonist (GW6471) alleviated CPF-induced behavioral deficits (1.80-fold increase in dark locomotion) and reduced elevated GABA and dopamine levels by 36.8% and 47.3%, respectively. Together, these results indicate that CPF can impair neuro-visual development and that oxidative stress and PPARα-related signaling are closely associated with these effects.

## 1. Introduction

The Food and Agriculture Organization (FAO) estimates that the global population will reach 10 billion by 2050, increasing the demand for food [1]. However, the food supply is challenged by climate change, agrochemical restrictions, plant diseases and pests [2]. Although agrochemical strategies have safeguarded food security to a certain extent, the application of pesticides poses risks to nontarget species [3]. Pesticides can contaminate aquatic ecosystems via spray drift, direct runoff, and leaching [4,5]. Pesticides are known to induce neurotoxicity through oxidative stress, neuroinflammation, and synaptic dysfunction [6]. Chlorpyrifos, an organophosphate pesticide, is widely used on food crops, cash crops, and oilseed crops to control a variety of pests [7]. Because of its extensive agricultural use and environmental persistence, chlorpyrifos has been frequently detected in rivers, lakes and coastal waters worldwide [8], and it shows moderate bioaccumulation in aquatic organisms [9]. The distribution of chlorpyrifos in surface water exhibits pronounced spatial heterogeneity, with detected concentrations ranging from the ng/L to the µg/L level [10]. In aquatic systems, chlorpyrifos residues tend to be detected at low levels (<0.1 µg/L). For instance, concentrations in the river sections of Guangzhou, China, and the Langat River in Malaysia have been recorded at 19 ng/L and 20 ng/L, respectively [10,11]. However, in regions influenced by intensive agricultural activities and surface runoff, concentrations can be magnitudes greater. Monitoring data from the Ethiopian Rift Valley watershed revealed a mean concentration as high as 0.38 µg/L, with peak levels reaching 8.0 µg/L [12], whereas specific extreme cases have documented values exceeding 40 µg/L [13].

Chlorpyrifos primarily affects the nervous system by blocking the active sites of acetylcholinesterase (AChE). Concentrations of chlorpyrifos ranging from 10 to 7500 μg/L inhibited cholinesterase activity in *Chilina gibbosa* in a dose–dependent manner [14]. Chlorpyrifos significantly inhibited acetylcholinesterase (AChE) in rainbow trout (*Oncorhynchus mykiss*) exposed to 1.38 μg/L [15], with 13 and 65 μg/L chlorpyrifos inducing developmental neurotoxicity and thyroid dysregulation in larval zebrafish (*Danio rerio*) [5]. Inland silversides (*Menidia beryllina*) dietarily exposed to chlorpyrifos-laden *Hyalella* presented a significant reduction in brain AChE [16]. In addition, the toxicity of chlorpyrifos has been associated with endocrine disorders, neural damage, oxidative stress, and behavioral effects. Head thrashing, body bending, crawling trajectories, and pharyngeal pumping were significantly altered in *Caenorhabditis elegans* following exposure to 50 μg/L chlorpyrifos [17]. Exposure to chlorpyrifos at concentrations as low as 0.05 μg/L for 72–96 h significantly increased wall-hugging behavior in delta smelt larvae, indicating anxiety responses or reduced exploration. At higher concentrations (up to 5 μg/L) and longer exposures, larvae showed locomotor disruption, including reduced cruising, increased burst and freezing behavior, and elevated trajectory complexity and rotation, reflecting impaired neural control and motor coordination [18]. Chlorpyrifos-oxon, the metabolic activation product of chlorpyrifos, can induce excessive ROS production via the activation of NADPH oxidase, thereby mediating neurotoxicity and ultimately leading to behavioral dysfunction [19]. In addition, recent research has revealed the non-AChE mechanisms of organophosphate-induced neurodevelopmental toxicity and immunotoxicity [20]. However, the mechanism by which chlorpyrifos causes behavior disorders at environmental concentrations remains to be elucidated.

Zebrafish are well-established model organisms commonly used in neurotoxicity studies because of their robust fecundity, in vitro fertilization, transparent embryos, rapid growth rate, and high genomic similarity to humans [21]. Locomotor behavior is a biological marker used when studying the effects of neurotoxic chemicals [22]. Larval zebrafish exhibited a 57% reduction in locomotion during dark periods when exposed to 141.88 mg/L thiacloprid [23]. A hyperactive response has also been reported for zebrafish exposed to 50 and 100 μg/L nano-formulated Cu(OH)_2_ pesticide (“nanopesticide”) during the dark phase [24]. This example is provided as prior context; nanoparticles/nanopesticides were not tested in the present study. The vertebrate visual system, especially the retina, is highly susceptible to these pathological processes due to its high metabolic demand and dependence on intact neural circuitry [25]. As an extension of the central nervous system, impairment of neural homeostasis can directly disrupt visual processing [26]. However, most studies on pesticide-induced neurotoxicity primarily assess general locomotor behavior or cholinergic dysfunction, while potential impacts on visual function remain largely unexplored [27]. Therefore, behaviour reliant upon intact visual processing may serve as a sensitive indicator of pesticide-induced neural damage [28]. Since the visual sensorimotor responses of zebrafish are detectable by 5 days post-fertilization (dpf), vision-guided locomotion behavioral assays may serve as a sensitive and underexplored indicator of neurotoxic effects on visual neural circuitry, particularly in response to light stimulation [20,21,29]. The zebrafish larval model was used for the present study. Accordingly, we hypothesized that short-term CPF exposure induces neuro-visual toxicity in early-life-stage zebrafish through oxidative stress and dysregulated PPARα signaling. To test this hypothesis, larvae were exposed to environmentally relevant CPF concentrations, and behavioral outcomes were integrated with neuronal fluorescence imaging in Tg (elavl3: EGFP) larvae, glial fibrillary acidic protein (GFAP) immunofluorescence, transcriptomics, targeted metabolomics, and bioinformatics analyses.

## 2. Materials and Methods

### 2.1. Chemicals and Reagents

Chlorpyrifos (CAS: 2921-88-2; molecular weight: 350.59; purity > 99%) was purchased from Aladdin Biochemical Technology (Shanghai, China). GW6471 (PPARα antagonist, CAS: 880635-03-0, purity > 98%), GSK0660 (PPARβ antagonist, CAS: 1014691-61-2, purity > 99%), and GW9662 (PPARγ antagonist, CAS: 22978-25-2, purity > 99%) were purchased from Bidepharm (Shanghai, China). Dimethyl sulfoxide (DMSO) was purchased from Sigma-Aldrich (St. Louis, MO, USA). All other chemicals and analytical grade reagents used in the experiment were purchased from Shanghai Hushi Laboratorial Equipment Co., Ltd. (Shanghai, China).

### 2.2. Zebrafish Rearing and Exposure

AB wild-type zebrafish and transgenic zebrafish Tg (*elavl3: EGFP*), which label postmitotic neurons with enhanced green fluorescence, were obtained from the China Zebrafish Resource Center (Wuhan, China). Zebrafish were reared in 40 L aerated aquariums at 28 ± 1 °C under a 14:10 h light/dark cycle. Adult zebrafish were fed three times a day with brine shrimp (*Artemia salina*) for one month to acclimate to the laboratory conditions. Males and females (1:1) were transferred into spawning tanks in the evening and bred the next morning. Embryos were collected within 2 h post-fertilization (hpf) and rinsed with 0.05% methylene blue solution. The embryos were kept in 150 mm culture glass dishes and incubated under the same conditions as the adults were, as described previously [30,31]. Considering that the retina of zebrafish is fully laminated by 5 dpf and that strong visually mediated behavioral responses can be detected by 6 dpf, 7 dpf zebrafish were used in this study to explore the relationship between chlorpyrifos and visual impairment [32]. Seven dpf larvae (200 per dish) were randomly distributed into 150 mm diameter glass culture dishes containing 200 mL of solution. According to our previous research, larvae were exposed to chlorpyrifos at concentrations of 0.01 μg/L (0.029 nM), 0.1 μg/L (0.29 nM), 1 μg/L (2.85 nM), 10 μg/L (28.5 nM), and 100 μg/L (285 nM) [33], with DMSO serving as the vehicle control (VC). 0.02% DMSO (*v*/*v*) was consistent across all treatment groups. The concentrations of chlorpyrifos in the exposure solutions were measured at 0 h and 24 h via UPLC–MS/MS (AB SCIEX, Marlborough, MA, USA). A 50 mL water sample was collected from each dish and stored at −20 °C until analysis, as detailed in Supporting Information S1. The average recoveries of the chlorpyrifos standards were in the range of 74.4–101.2% (Table S1). To determine whether PPAR signaling mediates chlorpyrifos-induced neurobehavioral and visual impairments, larvae were coexposed to chlorpyrifos (100 μg/L), a concentration previously shown to induce significant behavioral changes, together with selective PPAR antagonists: GW6471 (PPARα antagonist), GSK0660 (PPARβ antagonist), or GW9662 (PPARγ antagonist). Each group consisted of four biological replicates, and the number of zebrafish per replicate varied across different biological endpoints (e.g., behavioral assays, retinal histology, and gene expression). Following a 24 h exposure period, larvae were subjected to a series of endpoint assessments, including behavioral assays, transcriptomic analysis, neurotransmitter detection, retinal histological sectioning, and fluorescence-based measurements of ROS, Elavl3 protein and GFAP.

### 2.3. Photomotor and Strobe Response Assays

A photomotor response test and a strobe light test were used to assess visual function in larval fish [34]. A total of 48 larvae from each treatment group (0.01, 1, and 100 µg/L chlorpyrifos), and the vehicle control were used for the assays. For the photomotor response, zebrafish were transferred to 96-well plates and then placed into a DanioVision™ Chamber (Noldus, Wageningen, The Netherlands) to adapt to the dark for 10 min at room temperature (27 ± 2 °C); this acclimation period was not recorded and was excluded from subsequent analyses. The test started with light stimulation and was automatically recorded by EthoVision XT^®^ (Noldus, Wageningen, The Netherlands), which consisted of two cycles of 10 min of white light (“100% illumination”, 5000 lux) and 10 min of darkness (“0% illumination”, <1 lux). The distance (mm) was calculated per minute. The fish were then assessed for reactions with a strobe light test, which consisted of one cycle of 30 s of darkness and 30 s of strobe light. The distance (mm) was calculated per minute. The freezing index was calculated by the ratio of the distance (mm) traveled during the strobe light period to the distance (mm) traveled in the overall period.

### 2.4. Transcriptomics Analysis

For transcriptomic analysis, 80 larvae were randomly selected and pooled into four biological replicates (20 larvae per replicate). Total RNA was extracted using TRIzol reagent (Invitrogen Life Technologies, Carlsbad, CA, USA) after homogenization in liquid nitrogen. The RNA concentration and purity were assessed via a NanoDrop 2000 spectrophotometer (Thermo, Waltham, MA, USA), and RNA integrity was evaluated via an Agilent 2100 Bioanalyzer (Agilent, Santa Clara, CA, USA); only samples with an RNA integrity number (RIN) ≥ 7.0 were used for library preparation. RNA was extracted to prepare libraries, and RNA-seq libraries were constructed according to previously reported methodologies [3,5,35,36]. Sequencing was performed on an Illumina platform in paired-end 150 bp (PE150) mode, generating approximately 44–49 million paired-end reads per sample. The raw sequencing data was deposited in the NCBI database under accession number PRJNA1345080. Enrichment *p* values were calculated via the hypergeometric test, with a *p* value < 0.05 considered statistically significant. Differentially expressed genes (DEGs) were annotated with Kyoto Encyclopedia of Genes and Genomes (KEGG) enrichment analyses via topGO (v2.50.0) and clusterProfiler (v4.6.0), respectively. Ingenuity Pathway Analysis (IPA; Qiagen, Valencia, CA, USA) was used for predicting disease and function pathways based on DEGs. To validate the RNA-seq results, DEGs relevant to visual and nervous system functions were further examined via qRT–PCR. The detailed methods are provided in Supporting Information S2 and S3, and the primer sequences are listed in Table S2.

### 2.5. Neurotransmitter Metabolomics

Larvae were collected into centrifuge tubes (180 larvae, 45 pooled per 4 replicates) for neurotransmitter-targeted metabolomic assays. In brief, the samples were mixed with 500 μL of 70% methanol/water (*v*/*v*). The extracts were analyzed using an AB Sciex 6500^+^ QTRAP system (AB SCIEX, Framingham, MA, USA). Details are provided in the Supporting Information S4.

### 2.6. Neuronal Fluorescence Imaging and GFAP Immunofluorescence Analysis

Observations were conducted according to a previous study [30]. Briefly, 20 transgenic larvae of Tg (elavl3: EGFP) were randomly selected (n = 20 fish/group), and fluorescence images were captured with a Nikon M396C microscope (Nikon, Tokyo, Japan) using identical acquisition settings across groups. Fluorescence quantification was performed in ImageJ software [37]. For each larva, a consistent region of interest (ROI) encompassing the whole larval body (excluding the background) was outlined, and the mean gray value was measured after subtraction of the background signal measured from an adjacent nonfluorescent area. Fluorescence intensity values were normalized to the mean value of the vehicle control (VC) group and expressed as % of control.

GFAP is a marker of astrocytes, forms intermediate filaments in astrocytes and regulates their movement and shape [38], and the expression of GFAP in astrocytes was measured by immunofluorescence. Twenty larvae were randomly selected after exposure and fixed in 4% (*v*/*v*) paraformaldehyde at 4 °C overnight. The fish were embedded in paraffin and then sectioned at 20 µm using a microtome (Leica, Wetzlar, Germany). Sections were processed following standard immunofluorescence procedures (deparaffinization, antigen retrieval, blocking, and incubation with primary and fluorophore-conjugated secondary antibodies). Immunofluorescence staining was performed via an anti-GFAP pAb (GB11096, 1:2000, ServiceBio, Wuhan, China), and digital full-field slices were imaged with a scanning microscope (Pannoramic MIDI, 3DHISTECH, Budapest, Hungary). For quantification in ImageJ, anatomically matched ROIs (eye, brain, and olfactory bulb) were defined on comparable sections, and GFAP-positive fluorescence intensity (mean gray value after identical thresholding and background subtraction) was calculated and normalized to the VC group (% of control).

### 2.7. Retinal Sectioning and H&E Staining

Twenty larvae were fixed in 4% (*v*/*v*) paraformaldehyde at 4 °C overnight. Larvae were not fed throughout the exposure period prior to fixation. The samples were then dehydrated in graded alcohol and embedded in agarose using JB-P5 (Benor Medical Technology, Qingdao, China). The samples were cryoprotected and sectioned via a HistoCore BIOCUT cryostat (Leica Microsystems, Wetzlar, Germany) at a thickness of 10 µm, with the cryostat chamber temperature set to −18 °C. The cryosections were stained with hematoxylin for 5 min and then eosin for 5–8 s. Images were captured via an ECLIPSE Ni-U microscope (Nikon, Tokyo, Japan) and analyzed with ImageJ (v1.5) software.

### 2.8. AChE Activity and ROS Content Assessment

A total of 40 larvae were selected from each exposure group at random, with each group replicated four times (n = 4), with 10 larvae (7 dpf) collected from each replicate Petri dish. The AChE Assay Kit (Solarbio, Wuhan, China) was employed to determine the activity of the AChE following the manufacturer’s procedure. In brief, the fish were homogenized using an extract solution (pH 7.4) on ice and then centrifuged at 8000× *g* for 10 min at 4 °C. The supernatant was collected for measurement. In parallel, reactive oxygen species (ROS) levels were determined using a commercial ROS assay kit (Nanjing Jiancheng Bioengineering Institute, Nanjing, China) according to the manufacturer’s instructions. Both AChE activity and ROS levels were normalized to the wet weight of the samples.

### 2.9. Statistical Analysis

All of the experimental data are presented as the means ± standard error of the mean (SEM). Prior to parametric testing, normality of the data distribution was assessed using the Shapiro–Wilk test, and homogeneity of variance was assessed using Levene’s test. When assumptions were met, one-way analysis of variance (ANOVA) followed by the post hoc Sidak multiple comparison test was used to determine statistical significance between chlorpyrifos datasets. When normality or homoscedasticity assumptions were violated, the nonparametric Kruskal–Wallis test followed by Dunn’s multiple comparisons was applied. *p* value < 0.05 was considered statistically significant. Two-way ANOVA with Sidak’s post hoc test was used to compare the effects of chlorpyrifos exposure and co-exposure. Statistical analyses and figure preparation were performed with Prism 10 and Veusz (v3.3).

## 3. Results

### 3.1. Chlorpyrifos in Exposure Water

Chlorpyrifos was not detected in the vehicle control (VC) group. The concentrations of chlorpyrifos at 0 h were 0.1 ± 0.02, 0.9 ± 0.2, 10.3 ± 0.2 and 97.1 ± 10.2 μg/L in the 0.1, 1, 10 and 100 μg/L groups, respectively. At 24 h, the measured concentrations of chlorpyrifos were 0.09 ± 0.02, 0.8 ± 0.1, 9.4 ± 0.9 and 98.0 ± 9.5 μg/L in the chlorpyrifos groups, respectively (Table S3).

### 3.2. Chlorpyrifos-Induced Altered Photomotor Response

**Figure 1:**
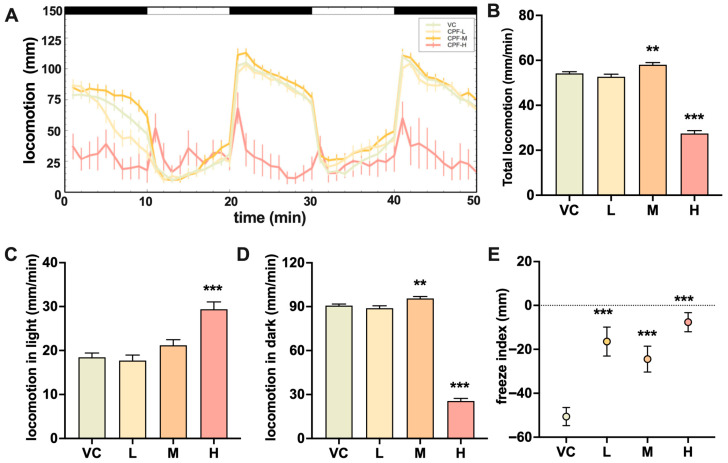
Effect of chlorpyrifos on (**A**) swimming distances in larvae per minute, (**B**) average overall distance, (**C**) average distance in the light period, (**D**) average distance in the dark period, and (**E**) freeze index in the strobe light test. In panel (**E**), the dashed line at 0 represents no difference between locomotion in light and dark periods. VC, vehicle control; Chlorpyrifos, CPF; L, M, and H indicate 0.01, 1, and 100 μg/L chlorpyrifos, respectively. 48 larvae were used from each treatment group. The error bars are presented as the SEM. Significant differences from VC are indicated with ** *p* < 0.01, and *** *p* < 0.001.

Larvae exposed to 100 μg/L chlorpyrifos exhibited a 48.90% decrease in overall swimming activity and a 57.71% decrease in swimming activity in the dark (Figure 1B,D) during the photomotor response assay, compared to the controls. No lethality or obvious gross morphological impairment was observed during the behavioral assay at this concentration, suggesting that the reduced locomotion was not attributable to moribundity. Larval activity inversely increased by 6.96% overall (*p* = 0.003) and by 5.40% (*p* = 0.011) in the dark (Figure 1D) in those exposed to 1 μg/L chlorpyrifos. As described in Section 2.3, the initial 10 min dark acclimation period was excluded from the analysis; the observed increase was primarily driven by the first scored dark segment of the photomotor cycles. In the strobe light assay, significant increases in the freezing index were observed in all the treatment groups (Figure 1E), compared to the controls.

### 3.3. Transcriptomic Effects Involved in Nervous and Visual System Function Following Chlorpyrifos Exposure

**Figure 2:**
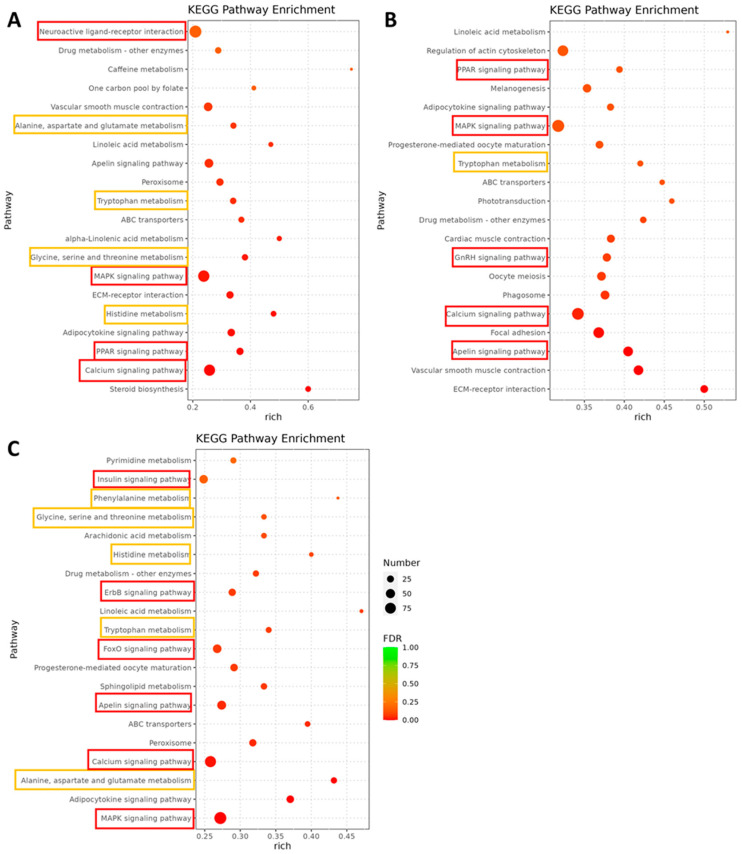
Top 20 enriched KEGG pathways following exposure to 0.01 (**A**), 1 (**B**), and 100 μg/L (**C**) chlorpyrifos. The pathways in the red rectangles are related to the nervous system, and the pathways in the yellow rectangles are related to neurotransmitter metabolism.

DEGs were identified by DESeq in the 0.01, 1, and 100 μg/L chlorpyrifos treatment groups (Figure S1). Functional enrichment analyses revealed prominent perturbations in nervous system function, eye development and visual function, and metabolism. KEGG enrichment analysis identified that the top enriched pathways were predominantly involved with nervous system and neurotransmitter metabolism, including GnRH signaling, apelin signaling, MAPK signaling, PPAR signaling, calcium signaling, and multiple amino acid metabolic pathways (alanine, aspartate, and glutamate metabolism; tryptophan metabolism; glycine, serine, and threonine metabolism; histidine metabolism; and phenylalanine metabolism) (Figure 2).

**Figure 3:**
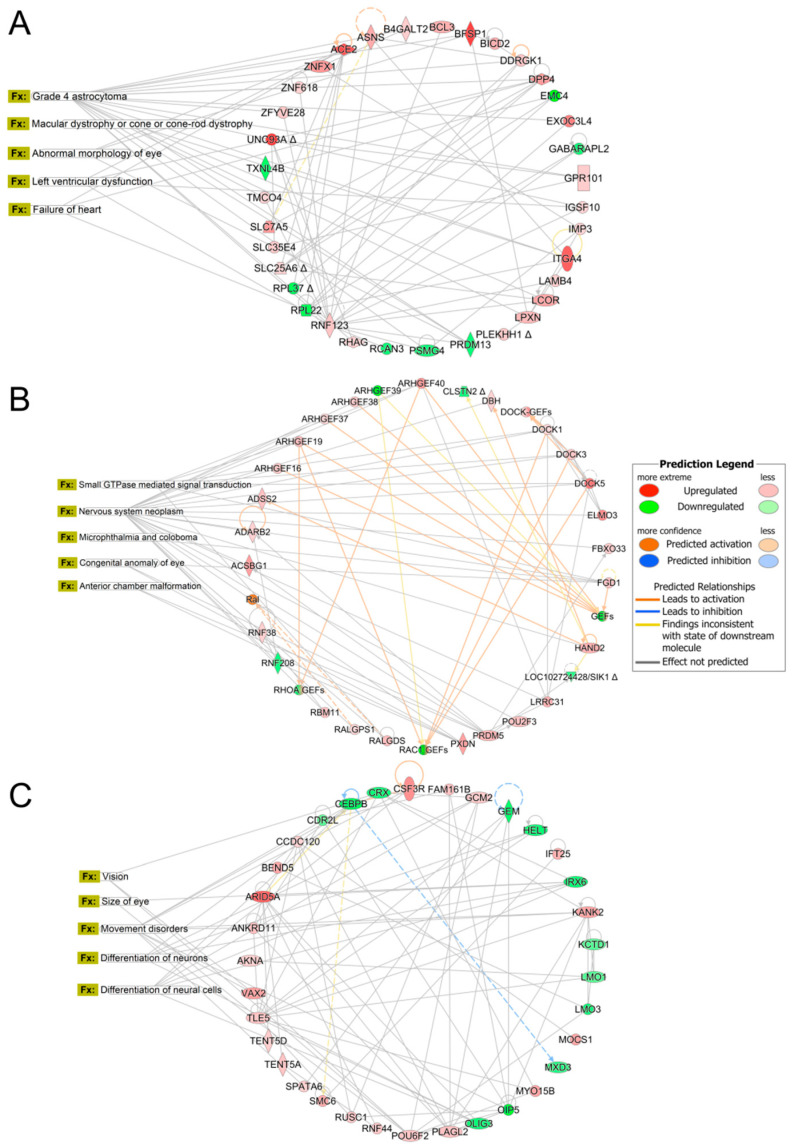
Top altered networks in Ingenuity Pathway Analysis involved in (**A**) cancer, neurological disease, and organismal injury and abnormalities in larvae exposed to 0.01 μg/L chlorpyrifos, (**B**) cell signaling, developmental disorder, and ophthalmic disease in larvae exposed to 1 μg/L chlorpyrifos, and (**C**) cancer, gene expression, and neurological disease in larvae exposed to 100 μg/L chlorpyrifos.

Organismal development, tissue morphology, organismal survival, digestive system development and function, and endocrine development were the top physiological system development and function pathways affected in larvae exposed to 0.01 μg/L chlorpyrifos (Table S4). The top altered network was involved in cancer, neurological disease, and organismal injury and abnormalities, with the top enriched diseases predicted to affect the incidence of astrocytoma, macular dystrophy or cone-rod dystrophy, abnormal morphology of eye, left ventricular dysfunction, and failure of heart in larvae exposed to 0.01 μg/L chlorpyrifos, with a score of 53 and 35 DEGs involved (Figure 3A).

Organismal survival, organismal development, tissue morphology, embryonic development, and nervous system development and function were the top physiological system development and function pathways affected in larvae exposed to 1 μg/L chlorpyrifos (Table S4). The top altered network was involved in cell signaling, developmental disorder, and ophthalmic disease, with the top enriched diseases predicted to affect small GTPase mediated signal transduction, nervous system neoplasm, microphthalmia, congenital anomaly of eye, and anterior chamber malformation of the eye in larvae exposed to 1 μg/L chlorpyrifos, with a score of 30 and 28 DEGs involved (Figure 3B).

Organismal survival, organismal development, tissue morphology, nervous system development and function, and digestive system development and function were the top physiological system development and function pathways affected in larvae exposed to 100 μg/L chlorpyrifos (Table S4). The top altered network was involved in cancer, gene expression, and neurological disease, with the top enriched diseases predicted to affect vision, size of eye, movement disorders, differentiation of neurons, and differentiation of neural cells in larvae exposed to 100 μg/L chlorpyrifos, with a score of 48 and 34 DEGs involved (Figure 3C). In line with the behavioral evidence suggesting impaired visual function, we further examined vision-related genes to validate transcriptomic profiling results. The normalized expression of *lamc1* and *ipo13* (visual perception/retinal development) increased, In contrast, genes enriched in the phototransduction pathway, such as *rlbp1a*, *rlbp1b* (phosphatidylinositol bisphosphate binding), *arl3l2* (GTP binding), *nyx* (oculomotor response), and *rho* (photoreceptor and rhodopsin signaling) decreased (Figure S2). Specifically, the downregulation of *rho* and *nyx* likely impairs the photomotor response, disrupting the organism’s ability to process light stimuli into locomotor activity. These expression patterns were validated by RT-qPCR, which showed a strong correlation with the RNA-seq data (Figure S3), confirming the reliability of the transcriptomic findings.

### 3.4. Chlorpyrifos Alters Neurotransmitter Levels

**Figure 4:**
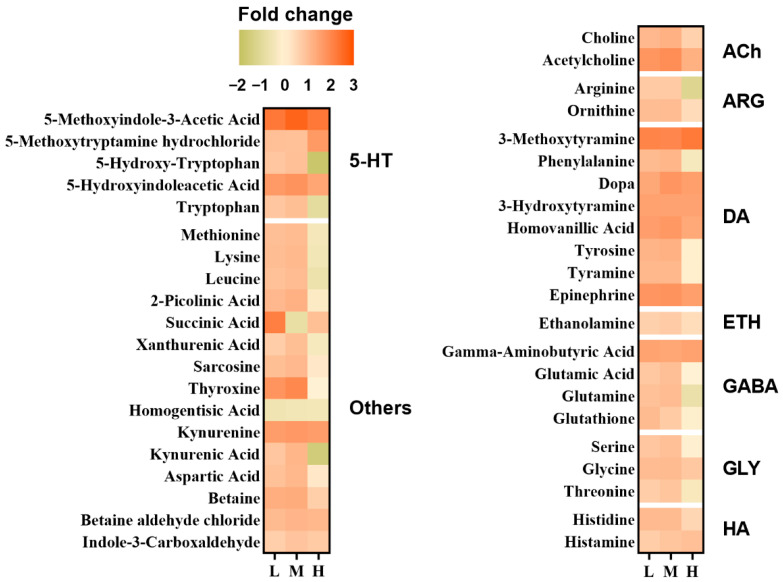
Heatmaps of acetylcholinergic (ACh), argininergic (ARG), dopaminergic (DA), ethanolaminergic (ETH), glutaminergic and GABAergic (GABA), glycinergic (GLY), histaminergic (HA), serotoninergic (5-HT), and other energetic neurotransmitters in zebrafish larvae exposed to chlorpyrifos. L, M, and H indicate 0.01, 1, and 100 μg/L chlorpyrifos, respectively. 180 larvae were used from each treatment group.

To verify the neurotoxic response and further identify the underlying molecular mechanisms, targeted metabolomic profiling was conducted. Among the 42 compounds quantified, 39 showed appreciable alterations following chlorpyrifos exposure (Figure 4). Notably, metabolites involved in neurotransmitter pathways, including dopaminergic, acetylcholinergic, serotonergic, glutamatergic, and GABAergic signaling, were markedly altered. Specifically, DOPA, 3-hydroxytyramine (DA), 3-methoxytyramine (3-MT), homovanillic acid (HVA), epinephrine (E), gamma-aminobutyric acid (GABA), 5-methoxyindole-3-acetic acid (5-MIAA), 5-hydroxyindoleacetic acid (5-HIAA), kynurenine, betaine aldehyde chloride, and acetylcholine (ACh) had consistently increased levels across all chlorpyrifos treatment groups (Table S5).

### 3.5. Chlorpyrifos Impaired Nervous, Astrocyte, and Retinal Development

**Figure 5:**
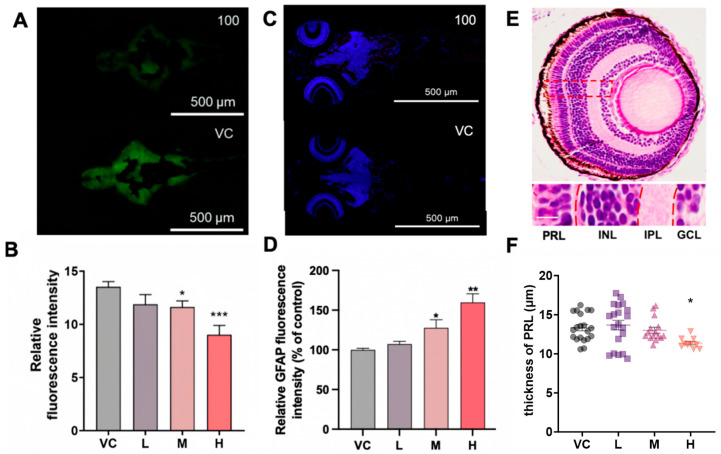
Effects of chlorpyrifos on zebrafish larval development at 8 dpf. (**A**) Fluorescence imaging of *Tg* (*elavl3: EGFP*) zebrafish. (**B**) Relative fluorescence intensity of *Tg* (*elavl3: EGFP*). (**C**) Immunofluorescence of GFAP. (**D**) Relative GFAP fluorescence intensity (% of control). (**E**) Retinal structure of larval zebrafish (bar = 10 μm). (**F**) Thickness of the PRL. PRL, photoreceptor layer; INL, inner nuclear layer; GCL, ganglion cell layer; IPL, inner plexiform layer; L, 0.01 μg/L chlorpyrifos; M, 1 μg/L chlorpyrifos; H, 100 μg/L chlorpyrifos. The error bars are presented as the SEM. 20 larvae were used from each treatment group. Significant differences are indicated with * *p* < 0.05, ** *p* < 0.01, and *** *p* < 0.001.

*Tg* (*elavl3: EGFP*) zebrafish enable the monitoring of neurogenesis; the relative fluorescence of elavl3 was significantly reduced by 12.1% and 32.5% in the 1 and 100 μg/L chlorpyrifos treatment groups, respectively (*p* < 0.001; Figure 5A,B). Astrocyte reactivity, assessed via GFAP immunohistochemistry, revealed a significant increase in GFAP-positive fluorescence within the eye, brain, and olfactory bulb following exposure to chlorpyrifos at both 1 μg/L (*p* = 0.037) and 100 μg/L (*p* = 0.002) (Figure 5C,D). Retinal development was further examined with histopathological assessment. The photoreceptor layer (PRL) thickness was significantly decreased by 14.3% in the 100 μg/L group (*p* = 0.034) (Figure 5E,F), and the inner nuclear layer (INL) and ganglion cell layer (GCL) were significantly reduced after 0.01 and 100 μg/L exposure (Figure S4A–C). Conversely, the inner plexiform layer (IPL) thickness increased by 21.7% following exposure to 1 μg/L chlorpyrifos (Figure S4D).

### 3.6. PPAR Antagonists Alleviated Larval Behavioral Disorders

**Figure 6:**
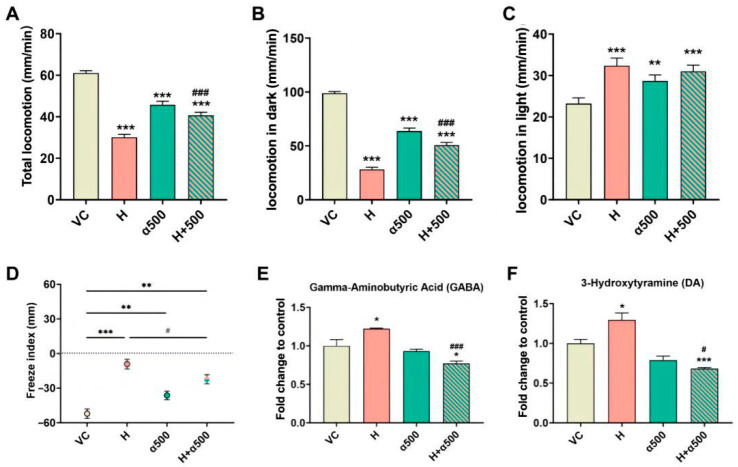
Effects of chlorpyrifos and GW6471 on photomotor behaviors and neurotransmitter levels in zebrafish larvae. (**A**) Average distances in the overall period. (**B**) Average distances in the dark period. (**C**) Average distances in the light period. (**D**) Freeze index in the strobe light test. (**E**) Levels of GABA. (**F**) DA levels. H indicates 100 μg/L chlorpyrifos, α500 indicates 500 μg/L GW6471, and H+α500 indicates coexposure to 500 μg/L GW6471 and 100 μg/L chlorpyrifos. In panel (**D**), the dashed line at 0 represents no difference between locomotion in light and dark periods. 48 larvae were used from each treatment group in the photomotor and strobe response assays and 180 larvae were used from each treatment group in the neurotransmitter assay. The error bars are presented as the SEM. Significant differences from VC are indicated with * *p* < 0.05, ** *p* < 0.01, and *** *p* < 0.001. Significant differences from H are indicated with ^#^ *p* < 0.05 and ^###^ *p* < 0.001.

The AChE activity (Figure S5) and ROS content (Figure S6) suggested that an oxidative stress–associated mechanism contributed to the observed neurobehavioral and visual impairments. In the omics analyses, KEGG enrichment and IPA predictions highlighted pathways linked to lipid metabolism/oxidative stress, including PPAR signaling (Figure 2 and Figure S7; Table S4). We therefore examined ROS and neurotransmitter levels and larval behaviors in the presence or absence of PPAR antagonists to better delineate mechanistic relationships. Larvae were coexposed to chlorpyrifos with GW6471 (a PPARα antagonist), GSK0660 (a PPARβ antagonist), or GW9662 (a PPARγ antagonist). Antagonism alone or coexposure did not affect zebrafish survival; because GW6471 alone produced a mild behavioral effect (Figure 6A–C), rescue was evaluated by comparing the coexposure group with both the chlorpyrifos-only and GW6471-only groups. Coexposure to GSK0660 or GW9662 failed to rescue chlorpyrifos-impaired locomotion in the dark (Figure S8A–F). In contrast, GW6471 was the most effective at alleviating chlorpyrifos-induced behavioral deficits: locomotion in the dark and total locomotion increased by 1.80- and 1.35-fold, respectively, compared with those in the 100 μg/L chlorpyrifos group (Figure 6A,B). Compared with those in the chlorpyrifos-exposed groups, a decrease in the levels of ROS was observed in the coexposure groups (Figure S9). GW6471 (500 μg/L) also partially mitigated the chlorpyrifos-induced alterations in the strobe light assay, although the response remained greater than that of the vehicle control (Figure 6D). Moreover, coexposure to 500 μg/L GW6471 reduced the GABA and DA levels by 36.80% and 47.34%, respectively (Figure 6E,F and Figure S10).

## 4. Discussion

Chlorpyrifos has been stringently regulated worldwide because of its environmental persistence and neurotoxicity, yet it is still frequently detected in surface waters [39], raising concerns about its potential threat to aquatic organisms during early developmental stages. Increasing evidence suggests that, at environmentally relevant concentrations, noncholinergic mechanisms may play a more decisive role in developmental neurotoxicity [40,41,42,43]. Behavioral assays, histopathology, transcriptomics, and targeted metabolomics were used in this study to elucidate the molecular basis of chlorpyrifos-induced abnormalities in neural and visual development in early life stage fish. Chlorpyrifos exposure at environmentally relevant concentrations (0.01 and 1 μg/L), as well as at a relatively high concentration (100 μg/L), elicited a pronounced biphasic behavioral response in larvae, accompanied by marked retinal remodeling and impaired neurogenesis. Chlorpyrifos-induced neuro-visual toxicity was strongly associated with a dysregulation in PPARα signaling. Rescue experiments with a PPARα-specific antagonist, GW6471, were conducted and support a strong role of PPARα in inducing hyperactivation, oxidative stress, and subsequent neural/visual injury imposed by chlorpyrifos, offering a new mechanistic insight for understanding the off-target toxicity of organophosphate pesticides.

Biphasic behavioral alterations induced by chlorpyrifos exposure were observed in this study. Elevated locomotor activity during the dark phase (0% illumination) is commonly interpreted as an anxiety-like response [44]. Larvae in the 1 μg/L chlorpyrifos group demonstrated a significant increase in distance traveled during the dark period, suggesting that low-dose chlorpyrifos may induce an anxiety-like state by perturbing neural circuits implicated in affective regulation, such as serotonergic or dopaminergic pathways [44]. Notably, low-dose stimulation involving excessive activity in natural settings could increase energy expenditure and potentially increase the risk of predation due to abnormal locomotion. Conversely, the decline in swimming activity observed in the 100 μg/L group likely signifies severe neurotoxic injury and/or exhaustion of energy reserves [45]. This phenotype corresponds to a nonmonotonic dose–response pattern [46], whereby low-level stressors can trigger compensatory responses, whereas higher levels of exposure can overwhelm physiological systems, culminating in functional collapse [47,48]. Furthermore, the presence of organophosphate has been demonstrated to induce sustained postsynaptic depolarization, which can subsequently result in neuromuscular transmission failure [49]. In addition, a strobe light assay revealed that exposure to chlorpyrifos led to a significant increase in the freeze index. This aberrant response to visual stimulation is indicative of impaired habituation or heightened sensitivity. This finding suggests that not only is motor dysfunction impaired but also deficits in visual information processing, which was among the top enriched pathways impaired, along with phototransduction. This, in turn, implicates the disruption of sensorimotor integration pathways following chlorpyrifos exposure [50].

Histopathological analysis revealed that exposure to chlorpyrifos substantially reduced the thickness of the PRL. The PRL is in the outer layers of the retina and is the primary site of phototransduction. The thinning of this layer can be indicative of photoreceptor loss and atrophy, which is consistent with retinal degenerative injury [51] and confirms transcriptomic pathway predictions. Concurrently, the INL and the GCL exhibited a marked reduction in thickness within the 0.01 and 100 μg/L groups. The INL comprises interneurons, including bipolar and amacrine cells, whereas the GCL signifies the final retinal output stage, which is responsible for transmitting visual signals to the brain [52]. The presence of concurrent damage across these layers suggests that chlorpyrifos toxicity is not confined to a single compartment but rather disrupts the retinal signaling cascade [53]. The IPL thickness markedly increased in the 1 μg/L group. As indicated by the presence of a synapse-rich zone between the INL and GCL, IPL thickening is indicative of early edema or compensatory synaptic remodeling. This finding is consistent with low-dose hyperactivity and suggests a transient pathological stage preceding overt neuronal loss [54]. The transcriptomic data further supported the hypothesis of functional impairment. Key vision-related genes, including *rho* (rhodopsin) [55], *nyx* (nyctalopin; photoreceptor–bipolar synaptic transmission in dim light) [56,57], and *rlbp1a/b* (CRALBP; visual cycle retinoid trafficking) [58,59], were downregulated. Conversely, the upregulation of *lamc1* and *ipo13* may serve as a marker for an injury-response involving extracellular matrix/basement membrane remodeling, potentially facilitated by activated Müller glia [60].

The application of targeted metabolomics was utilized to elucidate the disruption of neurotransmitters in larvae exposed to chlorpyrifos. In addition to the anticipated elevations in ACh, DA, GABA, E, and monoamine metabolites (HVA, 3-MT, and 5-HIAA), a general increase in these compounds was observed across all exposure groups. This implies a dysregulation of neurotransmitters rather than a purely cholinergic effect [61,62]. The concurrent increase in DA and its metabolites suggests stress-related remodeling of dopaminergic homeostasis [62,63], potentially involving compensatory tyrosine hydroxylase–linked synthesis [64] and impaired VMAT/DAT-mediated handling or clearance despite ongoing MAO activity [65], which could underlie low-dose hyperactivity. Simultaneously, the increase in GABA levels may represent a homeostatic response aimed at counteracting excessive excitatory activity to prevent neuronal damage [66]; however, simultaneous increases in ACh and GABA may result in the locking of circuits into a state with limited effective output, contributing to high-dose hypoactivity and exaggerated freezing to light [67].

Chlorpyrifos and chlorpyrifos-oxon may also disrupt lipid signaling and interfere with PPAR-related regulation through mechanisms that are not associated with cholinesterase targets [68,69,70]. The present study revealed that exposure to chlorpyrifos resulted in enrichment of the PPAR signaling pathway. PPARs, as members of the nuclear receptor superfamily, are widely involved in lipid metabolism and oxidative stress [71]. However, environmental pollutants have been shown to act as exogenous PPARα agonists, significantly activating this receptor pathway [72], and are closely linked to oxidative stress phenotypes [73]. The increased fatty acid oxidation driven by PPARα and the associated metabolic reprogramming in peroxisomes can increase the cellular ROS burden, thereby amplifying oxidative damage [74]. Consistent with these reports, the neurotoxic and visual impairments observed in this study are typical of oxidative stress pathology. The increased GFAP fluorescence in the eye and brain is indicative of reactive astrogliosis, a classic response of the central nervous system to oxidative damage and inflammatory stimuli [75,76]. Photoreceptor cells exhibit heightened sensitivity to oxidative stress, a phenomenon attributable to their elevated metabolic rate and high polyunsaturated fatty acid content. ROS have been shown to induce lipid peroxidation and apoptosis in the retina, which may explain why the retina is a primary target organ of toxicity [77]. Moreover, the kynurenine pathway, which is frequently activated under conditions of oxidative stress, involves neuroactive and potentially neurotoxic metabolites, providing a biological explanation for the increase in kynurenine observed in this study [78]. Rescue experiments with antagonists provide further confirmation of the central role of PPARα in chlorpyrifos-induced vision-guided behavioral disorders in fish. The PPARα antagonist, GW6471, which is a selective PPARα antagonist [79], demonstrated significant protective effects when coexposed with 100 μg/L chlorpyrifos. However, it is unclear why blocking PPARα, but not PPARβ or PPARγ, was so effective in reversing these effects.

## 5. Conclusions

The present study demonstrated that environmental concentrations of chlorpyrifos initiate the overactivation of PPARα, which can result in the generation of ROS, neuronal and retinal cell damage, neurotransmitter dysregulation, and behavioral abnormalities. These data further clarify the ecological effects of chlorpyrifos in aquatic environments. Future research is needed to verify whether similar PPARα-mediated mechanisms occur in higher vertebrate models and to investigate the detailed transcriptional crosstalk between PPARα, oxidative stress and neurotransmitter systems.

## Data Availability

The original contributions presented in this study are included in the article/Supplementary Materials. Further inquiries can be directed to the corresponding authors.

## Supplementary Materials

The following supporting information can be downloaded at: https://www.mdpi.com/article/10.3390/toxics14030234/s1. Information S1: Determination of Chlorpyrifos; Information S2: Transcriptomics Analysis; Information S3: RNA extraction and Quantitative Real-Time PCR Assay; Information S4: Neurotransmitter Metabolomics Analysis; Figure S1: Differential expression genes induced by chlorpyrifos; Figure S2: log2 fold change in vision-related genes following chlorpyrifos exposure; Figure S3: Correlation analysis of RNA sequencing (RNA-Seq) and qRT-PCR results; Figure S4: Structural changes in the retina of zebrafish larvae; Figure S5: Effects of chlorpyrifos on acetylcholinesterase activity; Figure S6: Changes in reactive oxygen species levels following chlorpyrifos exposure; Figure S7: IPA analysis of molecules regulating central nervous system morphology in zebrafish larvae exposed to chlorpyrifos; Figure S8: Effects of chlorpyrifos and PPAR antagonists on vision-guided behaviors and neurotransmitter levels; Figure S9: Effects of chlorpyrifos and GW6471 coexposure on reactive oxygen species levels; Figure S10: Heatmap of neurotransmitter alterations in zebrafish larvae following chlorpyrifos exposure; Figure S11: The CT values of β-actin in zebrafish exposed to chlorpyrifos; Table S1: Recoveries and relative standard deviations of chlorpyrifos in water samples; Table S2: Primers used for qRT-PCR validation; Table S3: Measured aqueous concentrations of chlorpyrifos during exposure; Table S4: Top five enriched physiological system development and function pathways predicted in Ingenuity Pathway Analysis; Table S5: Quantitative data of chlorpyrifos in targeted neurotransmitter metabolomics analyses.
