# Supplementary material for: PPARα Antagonism Rescues Chlorpyrifos-Induced Neuro-Visual Toxicity in Zebrafish (Danio rerio) Larvae
Source: Toxics. 2026 Mar 9;14(3):234. doi: 10.3390/toxics14030234 (PMC13029991; doi:10.3390/toxics14030234)
Supplement: Supplementary file 1 [file toxics-14-00234-s001.zip › toxics-4152041-supplementary.pdf]

Supporting information

PPARα antagonism rescues chlorpyrifos-induced neuro-visual toxicity in zebrafish (Danio rerio) larvae

Yuyao Jiang [a #], Zijie Ding [a #], Ruolin Hu [a], Jason T. Magnuson [c], Shiyan Li [d], Dingnan Wang [d], Shengli Zhou [e,]*, Yirong Guo [a], Yang Wang [d], Yuanyuan Liu [a], Shuying Li [a,b,]*, Wenjun Gui [a]

[a] Institute of Pesticide and Environmental Toxicology, College of Agriculture and Biotechnology, Zhejiang University, Hangzhou 310058, P. R. China

[b] Zhejiang Key Laboratory of Biology and Ecological Regulation of Crop Pathogens and Insects, Ministry of Agriculture Key Laboratory of Molecular Biology of Crop Pathogens and Insect Pests, Zhejiang University, Hangzhou 310058, P. R. China

[c] U.S. Geological Survey, Columbia Environmental Research Center, Columbia, MO 65201, USA

[d] Zhejiang Fisheries Technical Extension Center, Hangzhou 310023, P. R. China

[e] Ecological and Environmental Monitoring Center of Zhejiang Province, Hangzhou 310012, P. R. China

Author Information

Corresponding Author

Shengli Zhou- Ecological and Environmental Monitoring Center of Zhejiang Province, Hangzhou 310012, P. R. China

Email: slzhou2002@163.com

Shuying Li - Institute of Pesticide and Environmental Toxicology, Zhejiang University, Hangzhou, 310058, P. R. China, Hangzhou 310058, P. R. China

Email: lishuyingabc@zju.edu.cn

Supporting Information (SI-1)

Determination of Chlorpyrifos

For water samples, 20 mL of each sample was put into a 50 mL centrifuge tube. Then, 20 mL of acetonitrile, 1 g of NaCl and 3 g of $MgSO_4$ were added, and the mixture was hand-shaken for 1 min. Afterward, the tube was centrifuged at 4000 rpm for 10 min, and 10 mL of the supernatant was collected. The organic phase was concentrated with nitrogen at 40℃ until almost dry. The residue was recovered with 1 mL of acetonitrile and filtered through a 0.22 μm PTFE filter for UPLC-MS/MS analysis.

The UPLC-MS/MS instrument was composed of a 5500 QTRAP MS/MS system (AB SCIEX, Singapore) and a Waters ACQUITY™ system (Waters, Milford, USA). An ACQUITY UPLC® HSS T3 column (1.8 μm particle size, 100 mm × 2.1 mm i.d., Waters, USA) was used. The temperature of the column oven was set at 40 ℃, and the injection volume was 2 μL. The mobile phases were used at a flow rate of 0.3 mL/min. Solvent A was composed of water containing 0.1% formic acid, and solvent B was acetonitrile. The proportions were as follows: 80% A (from 0 to 8 min), 5% A (8 to 10 min), 80% A (10 to 10.1 min) and 80% A (until 14 min). The ESI source in positive mode was set as follows: ion spray (IS) voltage, 5500 V; nitrogen collision gas (CAD), 5 psi; curtain gas, 30 psi; nebulizer gas (GS1), 30 psi; auxiliary gas (GS2), 30 psi; source temperature, 350 ℃; declustering potential (DP): 50.0 V; entrance potential (EP): 3.0 V; and collision energy (CE): 45.5 eV and 45.5 eV. Multiple reaction monitoring was then performed with the most intense channel at m/z 350.0 > 198.0 used for quantification and m/z 350.0 > 97.0 used for confirmation.

Supporting Information (SI-2)

Transcriptomics Analysis

Total RNA was isolated using the Trizol Reagent (Invitrogen Life Technologies), after which the concentration, quality and integrity were determined using a NanoDrop spectrophotometer (Thermo Scientific). Three micrograms of RNA were used as input material for the RNA sample preparations. Sequencing libraries were generated according to the following steps. Firstly, mRNA was purified from total RNA using poly-T oligo-attached magnetic beads. Fragmentation was carried out using divalent cations under elevated temperature in an Illumina proprietary fragmentation buffer. First-strand cDNA was synthesized using random oligonucleotides and SuperScript II. Second-strand cDNA synthesis was subsequently performed using DNA Polymerase I and RNase H. Remaining overhangs were converted into blunt ends via exonuclease/polymerase activities and the enzymes were removed. After adenylation of the 3′ends of the DNA fragments, Illumina PE adapter oligonucleotides were ligated to prepare for hybridization. To select cDNA fragments of the preferred 400-500 bp in length, the library fragments were purified using the AMPure XP system (Beckman Coulter, Beverly, CA, USA). DNA fragments with ligated adaptor molecules on both ends were selectively enriched using Illumina PCR Primer Cocktail in a 15-cycle PCR reaction. Products were purified (AMPure XP system) and quantified using the Agilent high-sensitivity DNA assay on a Bioanalyzer 2100 system (Agilent). The sequencing library was then sequenced on NovaSeq 6000 platform (Illumina).

Supporting Information (SI-3)

RNA Extraction and Quantitative Real-Time PCR (qRT-PCR) Assay

Larval samples were collected and preserved with TRIzol reagent for obtaining RNA. The content of RNA was measured by the NanoDrop spectrophotometer (NanoDrop Technologies Inc., Wilmington, DE) and the quality was determined by a 260/280 nm absorbance ratio between 1.8 - 2.0. First-strand cDNA synthesis was performed using a PrimeScript RT reagent Kit with gDNA Eraser (Takara, China) following the manufacturer's instructions. The qRT-PCR was conducted on ABI StepOnePlus™ Real-Time PCR System (PerkinElmer Applied Biosystems, USA) to examine the selected genes (*rho, nyx, arl3l2, rlbp1b, rlbp1a, stra6l, ipo13, lamc1*), and the primers of those genes were assessed and are listed in Table S1. *β*-actin as a housekeeping gene was selected to normalize mRNA expression, which was stable during exposure (Figure S11). The mRNA expression level was calculated using the $2^{-\triangle\triangle Ct}$ method, with 2-well technical replicates and 4 biological replicates.

Supporting Information (SI-4)

Neurotransmitter Metabolomics.

After the sample was thawed and smashed, 0.05 g of the sample was mixed with 500 μL of 70% methanol/water. The sample was vortexed for 3 min under the condition of 2500 r/min and centrifuged at 12000 r/min for 10 min at 4°C. A total of 300 μL of the supernatant was poured into a new centrifuge tube and placed in a refrigerator at -20°C for 30 min. Then, the supernatant was centrifuged again at 12000 r/min for 10 min at 4°C. After centrifugation, 200 μL of the supernatant was transferred for further LC-MS analysis.

The sample extracts were analyzed using an LC-ESI-MS/MS system (UPLC, ExionLC; MS, QTRAP® 6500+ System, https://sciex.com /). The analytical conditions were as follows, HPLC: column, Waters ACQUITY UPLC HSS T3 C18 (100 mm×2.1 mm i.d.ꞁ1.8 μm); solvent system, water with 0.1% formic acid (A), acetonitrile with 0.1% formic acid (B); the gradient was started at 5% B (0 min), increased to 95% B (0-8 min), 95% B (8-9.5 min), and finally ramped back up to 5% B (9.6-12 min); flow rate, 0.35 mL/min; temperature, 40°C; injection volume: 2 μL. AB 6500+ QTRAP® LC-MS/MS System, equipped with an ESI Turbo Ion-Spray interface, operating in both positive- and negative-ion modes and controlled by Analyst 1.6 software (AB Sciex). The ESI source operation parameters were as follows: ion source, turbo spray; source temperature 550°C; ion spray voltage (IS) 5500 V^Positive∨,-4500 V(Negative); curtain gas (CUR) was set at 35.0 psi; DP and CE for individual MRM transitions were achieved with further DP and CE optimization. A specific set of MRM transitions was monitored for each period according to the neurotransmitters eluted within this period.

TABLES

Table S1. Recoveries and RSDs of the chlorpyrifos samples in water (n=4).

Spiked levels	0.1 μg/L		10 μg/L		100 μg/L	
Chlorpyrifos	Recoveries (%)	RSDs (%)	Recoveries (%)	RSDs (%)	Recoveries (%)	RSDs (%)
	70.5	19.7	113.4	15.7	108.2	5.6

Table S2. Primers used for qRT-PCR validation.

Gene	Sequence of the Primer (5'-3')	Sequence of the Primer (3'-5')
rho	GGTCGCTTGTAGTACTGGC	ATGTAACGCGACCAGCC
nyx	GGACTGTTCGGTGCTTCTCA	TCTCCAGCTTCATGTGCCAG
arl3l2	ATGGATGATGAAATTGCCGC	AGTTGGTGACAATACCGTGC
rlbp1b	CTGCGTGCCTACTGTGTAATCC	ATCACATGCACAGCCTTAAACC
rlbp1a	GTCAAACCCCTGATGAAGAGC	GTGATCTTGCCGTCATATTTGG
stra6l	GGTCCATGGTCCTGCACAGG	CAATCTTCAGGTAATGGGAG
ipo13	GTATGAAAGCCTAAAGGCACAGC	GCCGAGTCAGTACAATCTTGGAG
lamc1	ACCTGACCAGCTTACCTCCA	CAGCCGTCTGTTGCTCAGTT
β-actin	CGAGCAGGAGATGGGAACC	CAACGGAAACGCTCATTGC

Table S3. Measured aqueous concentrations of chlorpyrifos during exposure.

	Nominal Concentration (μg/L)	Average Measured Concentration (μg/L)	
		0 h	24 h
Chlorpyrifos	VC	ND	ND
	0.01	< LOD	< LOD
	0.1	0.1 ± 0.02	0.09 ± 0.02
	1	0.9 ± 0.2	0.8 ± 0.1
	10	10.3 ± 0.2	9.4 ± 0.9
	100	97.1 ± 10.2	98.0 ± 9.5

Values are represented as mean ± standard deviation (n=4). ND = not detected. The limit of quantitation method of chlorpyrifos was 0.03 μg/L. LOD = limit of detection. VC = vehicle control.

Table S4. Top five enriched physiological system development and function pathways predicted in Ingenuity Pathway Analysis.

Treatment	Pathway	Functional annotation	p-value
0.01 µg/L	Organismal development	Morphology of body cavity	1.23E-20
	Tissue morphology	Quantity of cells	5.92E-17
	Organismal survival	Organismal death	3.79E-16
	Digestive system development and function	Morphology of digestive system	9.07E-15
	Endocrine system development and function	Concentration of hormone	4.50E-14
1 µg/L	Organismal survival	Organismal death	7.31E-33
	Organismal development	Morphology of body cavity	4.80E-27
	Tissue morphology	Quantity of cells	1.69E-22
	Embryonic development	Development of head	5.11E-19
	Nervous system development and function	Morphology of nervous system	3.16E-18
100 µg/L	Organismal survival	Organismal death	8.21E-22
	Organismal development	Morphology of body cavity	3.36E-19
	Tissue morphology	Quantity of cells	8.65E-16
	Nervous system development and function	Morphology of nervous system	3.46E-15
	Digestive system development and function	Morphology of digestive system	4.15E-15

Table S5. Quantitative data of chlorpyrifos in the targeted neurotransmitter metabolomics analyses (n=4), indicating the effects of the chlorpyrifos on the tested neurotransmitters in early-stage zebrafish. The numbers were the log2 fold change of the treated mean to the control mean. L= 0.01 μg/L chlorpyrifos, M = 1 μg/L chlorpyrifos, H = 100 μg/L chlorpyrifos. (*) represents a log2 fold change < -1 or > 1.

	Metabolites	L/VC	M/VC	H/VC
Acetylcholinergic (ACh)	Choline	1.07*	1.22*	0.61
	Acetylcholine	1.72*	1.88*	1.23*
Argininergic (ARG)	Arginine	0.74	0.75	-1.22*
	Ornithine	0.99	1.02*	0.42
Dopaminergic (DA)	3-Methoxytyramine	2.06*	2.01*	2.26*
	Phenylalanine	1.02*	1.12*	-0.40
	Dopa	1.39*	1.73*	1.55*
	3-Hydroxytyramine	1.51*	1.49*	1.48*
	Homovanillic Acid	1.57*	1.68*	1.38*
	Tyrosine	1.15*	1.22*	-0.15
	Tyramine	1.10*	1.10*	-0.17
	Epinephrine	1.73*	1.77*	1.55*
Ethanolaminergic (ETH)	Ethanolamine	0.62	0.71	0.39
Glutaminergic and GABAergic (GABA)	Gamma-Aminobutyric Acid	1.48*	1.42*	1.51*
	Glutamic Acid	0.78	0.94	0.01
	Glutamine	0.91	1.00*	-0.76
	Glutathione	1.03*	0.72	-0.19
Glycinergic (GLY)	Serine	0.82	0.93	0.06
	Glycine	1.00*	1.02*	0.78
	Threonine	0.69	0.84	-0.44
Histaminergic (HA)	Histidine	1.03*	1.03*	0.51
	Histamine	0.67	0.82	0.92
Serotoninergic (5-HT)	5-Methoxyindole-3-Acetic Acid	2.29*	2.63*	2.27*
	5-Methoxytryptamine hydrochloride	0.92	0.92	1.64*
	5-Hydroxy-Tryptophan	0.81	0.93	-1.84*
	5-Hydroxyindoleacetic Acid	1.64*	1.78*	1.44*
	Tryptophan	0.82	0.95	-0.97
Other-energetic signaling	Methionine	0.94	1.01*	-0.47
	Lysine	0.99	1.07*	-0.55
	Leucine	0.92	1.02*	-0.73
	2-Picolinic Acid	1.07*	1.22*	-0.34

Succinic Acid	2.16*	-0.87	0.94
Xanthurenic Acid	0.67	0.96	-0.41
Sarcosine	0.95	1.08*	0.19
Thyroxine	1.74*	1.97*	-0.03
Homogentisic Acid	-0.61	-0.55	-0.53
Kynurenine	1.59*	1.67*	1.59*
Kynurenic Acid	0.81	1.12*	-1.60*
Aspartic Acid	0.90	1.09*	0.21
Betaine	1.29*	1.32*	0.65
Betaine aldehyde chloride	1.02*	1.14*	1.10*
Indole-3-Carboxaldehyde	0.64	0.85	0.73

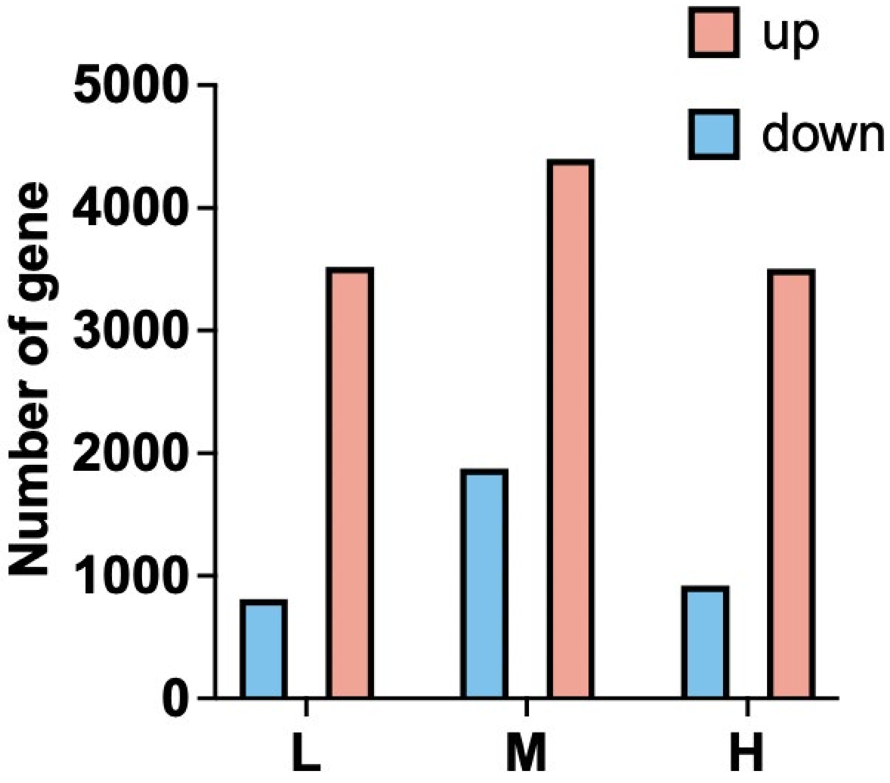

Figure S1. Differential expression genes induced by chlorpyrifos. The columns in red represent up-regulated DEGs compared to the control, and the columns in white represent down-regulated DEGs compared to the control. L, M, and H indicate 0.01, 1, and 100 μg/L chlorpyrifos, respectively.

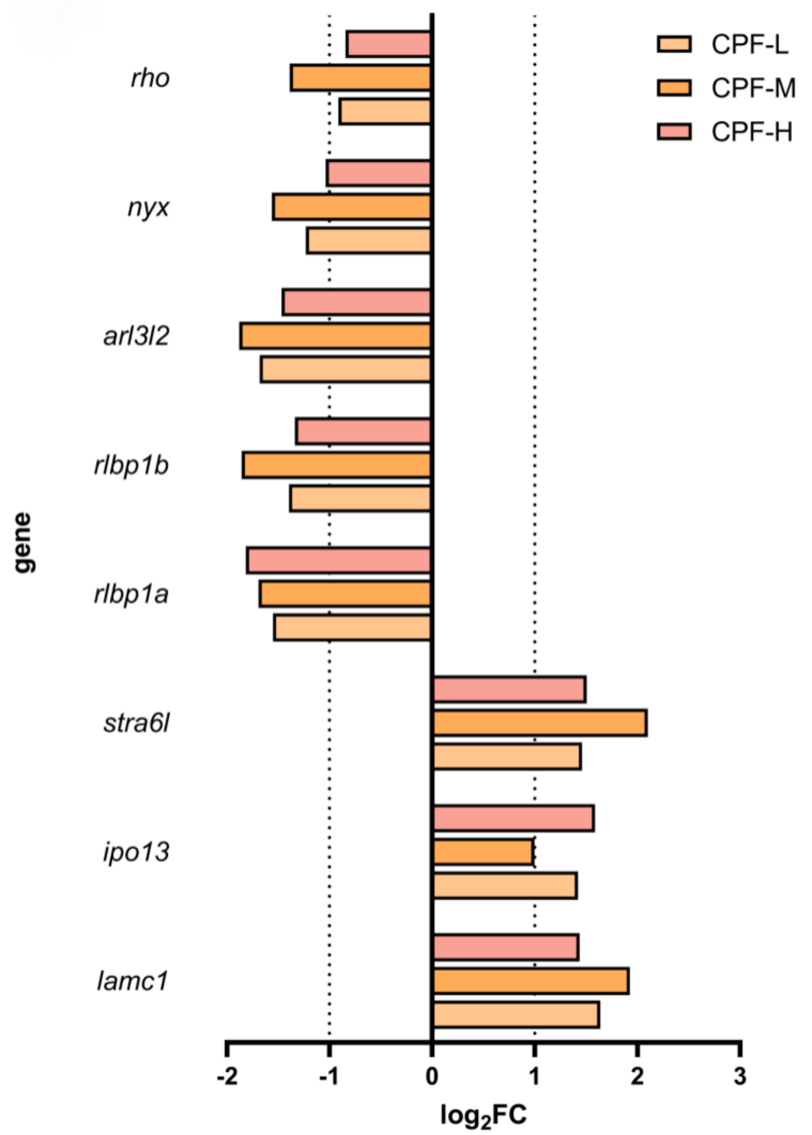

Figure S2. Log$_2$ fold change (log$_2$ FC) in vision-related genes following CPF exposure. The x-axis represents the log$_2$-transformed fold changes in gene expression, and the y-axis indicates the names of vision-related genes. CPF-L, CPF-M, and CPF-H denote the 0.01, 1, and 100 μg/L CPF exposure groups, respectively.

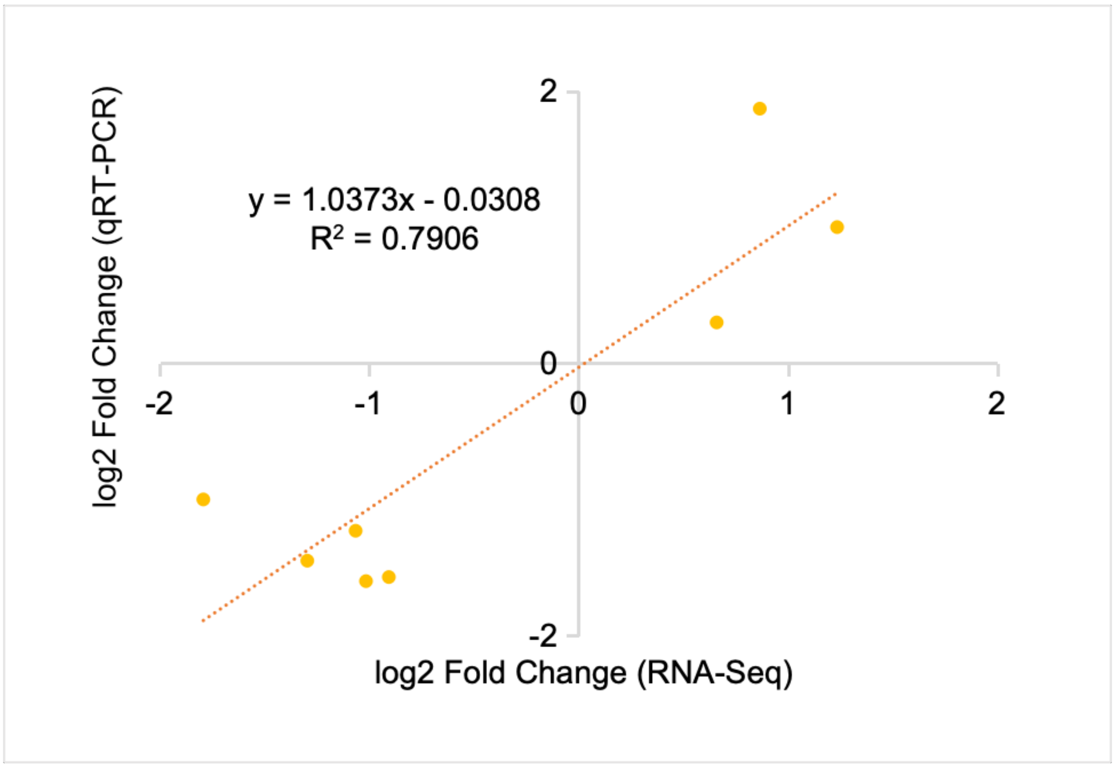

Figure S3. Correlation analysis of RNA sequencing (RNA-Seq) and qRT-PCR results (linear fitting method)

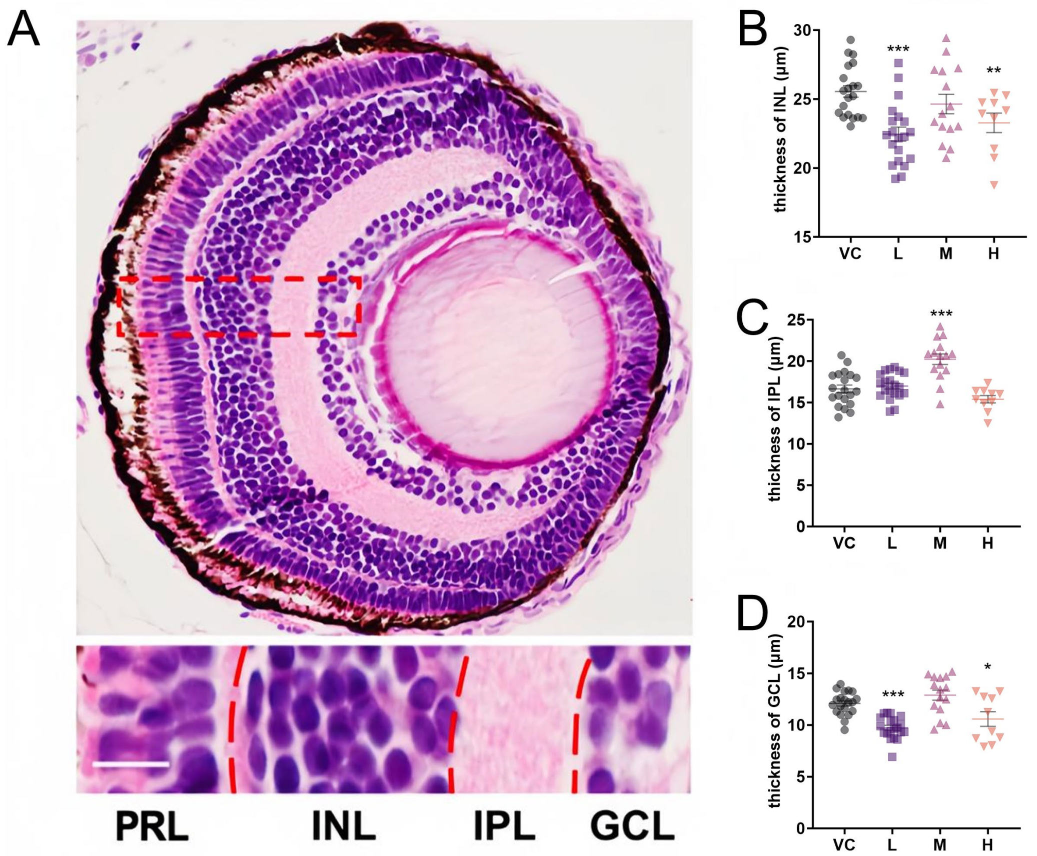

Figure S4. Structural changes in the retina of zebrafish larvae. (A) Structure of the retina of larval zebrafish (bar = 10 μm). Statistical analysis of the thickness of INL (B), IPL (C), and GCL (D). Abbreviation: INL, inner nuclear layer; IPL, inner plexiform layer; GCL, ganglion cell layer; L, 0.01 μg/L chlorpyrifos; M, 1 μg/L chlorpyrifos; H, 100 μg/L chlorpyrifos. The error bars are presented as the SEM. Significant differences are indicated with **p* < 0.05, ***p* < 0.01 and ****p* < 0.001.

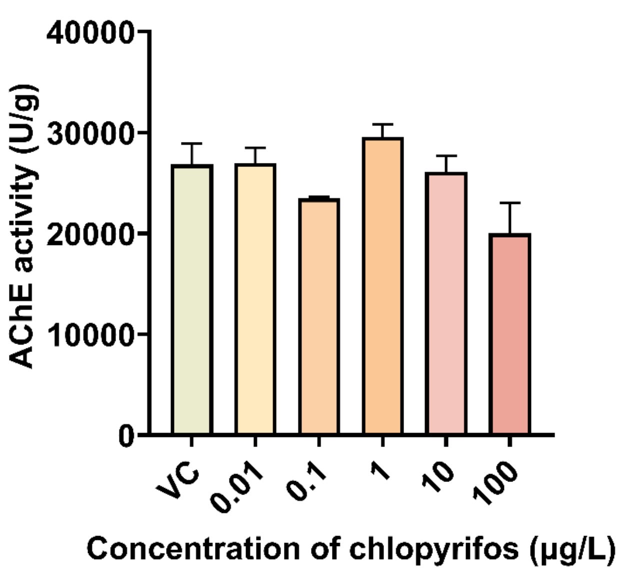

Figure S5. Effects of chlorpyrifos on AChE activity in zebrafish (*Danio rerio*). Error bars are presented as the standard error mean.

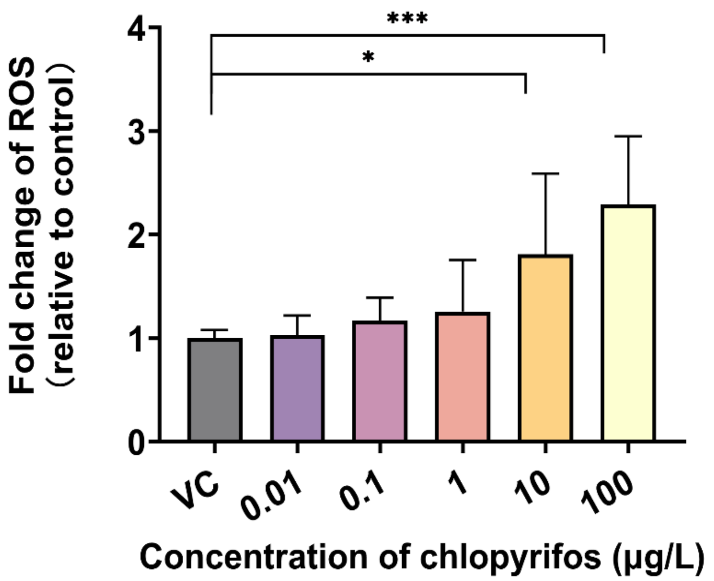

Figure S6. Changes in reactive oxygen species (ROS) levels following exposure to different concentrations of chlorpyrifos (CPF). The y-axis represents the fold change in ROS relative to the vehicle control (VC), and the x-axis indicates CPF concentrations (µg/L). Data are presented as mean ± SEM. Compared with the VC group, CPF exposure induced a dose-dependent increase in ROS levels, with significant elevations observed at 10 and 100 µg/L. * $p < 0.05$; *** $p < 0.001$.

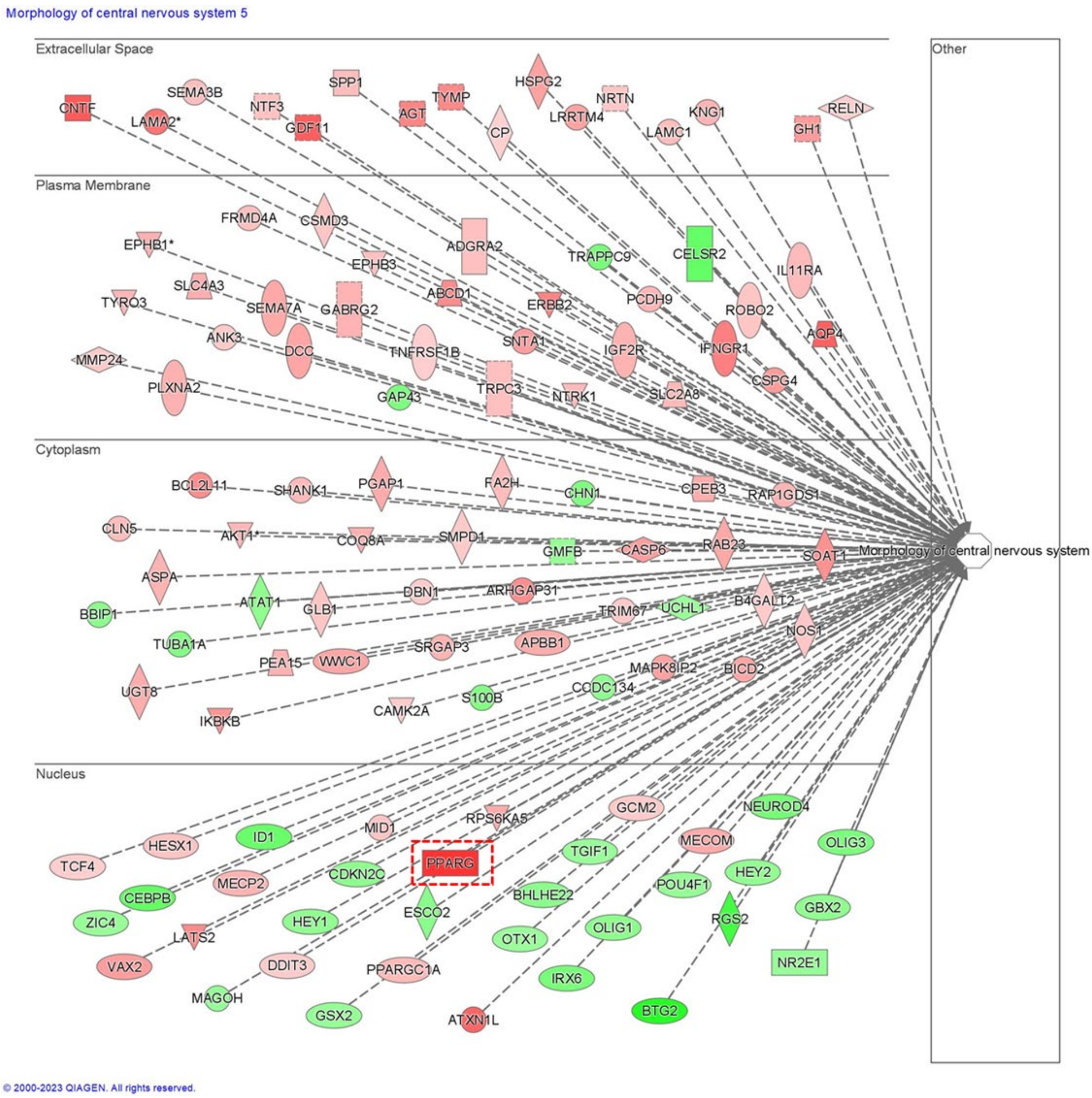

Figure S7. IPA analysis in zebrafish larvae exposed to chlorpyrifos. Molecules predicted to regulate the morphology of the central nervous system. The genes in green and red represent the significantly down- or up-regulated DEGs after chlorpyrifos exposure, respectively.

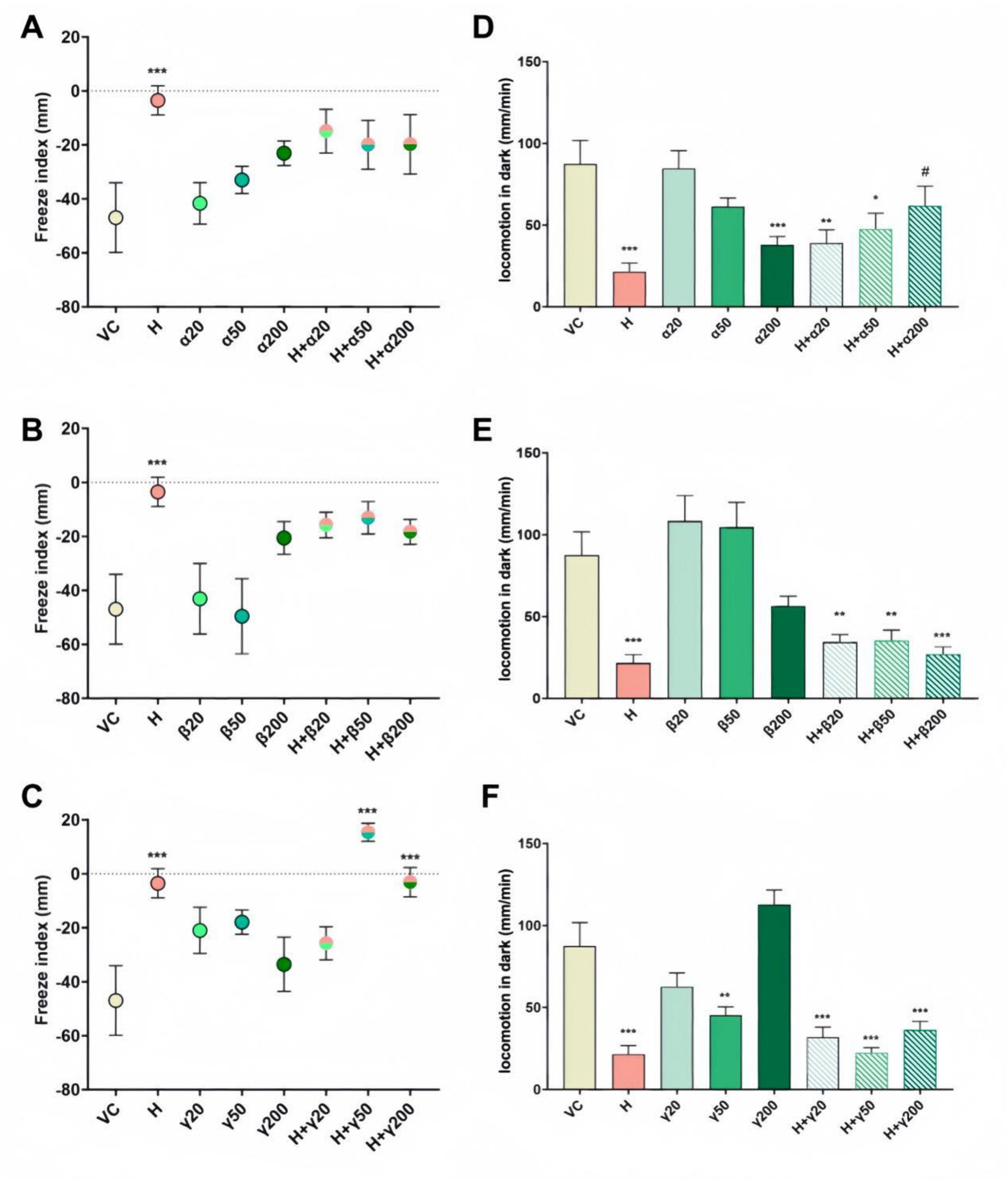

Figure S8. Impacts of chlorpyrifos and PPAR antagonists on vision-guided behaviors and neurotransmitter levels of zebrafish larvae. (A) Freeze index in strobe light test following coexposure with chlorpyrifos and PPARα antagonist GW6471. (B) Freeze index in strobe light test following coexposure with chlorpyrifos and PPARβ antagonist GSK0660. (C) Freeze index in strobe light test following coexposure with chlorpyrifos and PPARγ antagonist GW9662. (D) Average distances in the dark period following coexposure with chlorpyrifos and PPARα antagonist GW6471. (E) Average distances in the dark period following coexposure with chlorpyrifos and PPARβ antagonist GSK0660. (F) Average distances in the dark period following coexposure with chlorpyrifos and PPARγ antagonist GW9662. H indicated 100 μg/L chlorpyrifos, α20 indicated 20 μg/L GW6471, H+α20 indicated coexposure 20 μg/L GW6471 with 100 μg/L

chlorpyrifos. β20 indicated 20 μg/L GW6471, H+β20 indicated coexposure 20 μg/L GSK0660 with 100 μg/L chlorpyrifos. γ20 indicated 20 μg/L GW6471, H+γ20 indicated coexposure 20 μg/L GW9662 with 100 μg/L chlorpyrifos. Error bars are presented as the SEM. Significant differences to VC are indicated with * $p < 0.05$, ** $p < 0.01$ and *** $p < 0.001$. Significant differences to H are indicated with # $p < 0.05$.

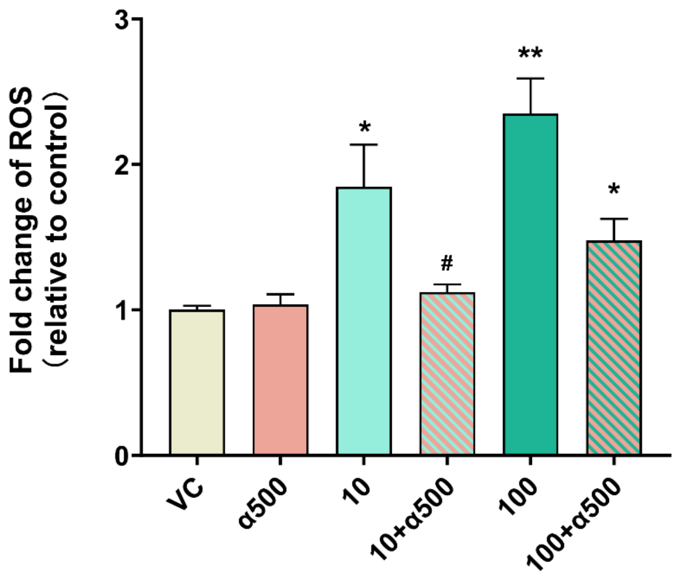

Figure S9. Effects of chlorpyrifos (CPF) exposure and GW6471 coexposure on reactive oxygen species (ROS) levels. The y-axis represents the fold change in ROS relative to the vehicle control (VC). Larvae were exposed to CPF alone (10 or 100 μg/L), GW6471 alone (α500; 500 μg/L), or coexposed to CPF and GW6471 (10 + α500 or 100 + α500). Data are presented as mean ± SEM. CPF exposure significantly increased ROS levels, whereas coexposure with GW6471 partially attenuated CPF-induced ROS elevation. * $p < 0.05$, ** $p < 0.01$ compared with VC; # $p < 0.05$ compared with the corresponding CPF-only group.

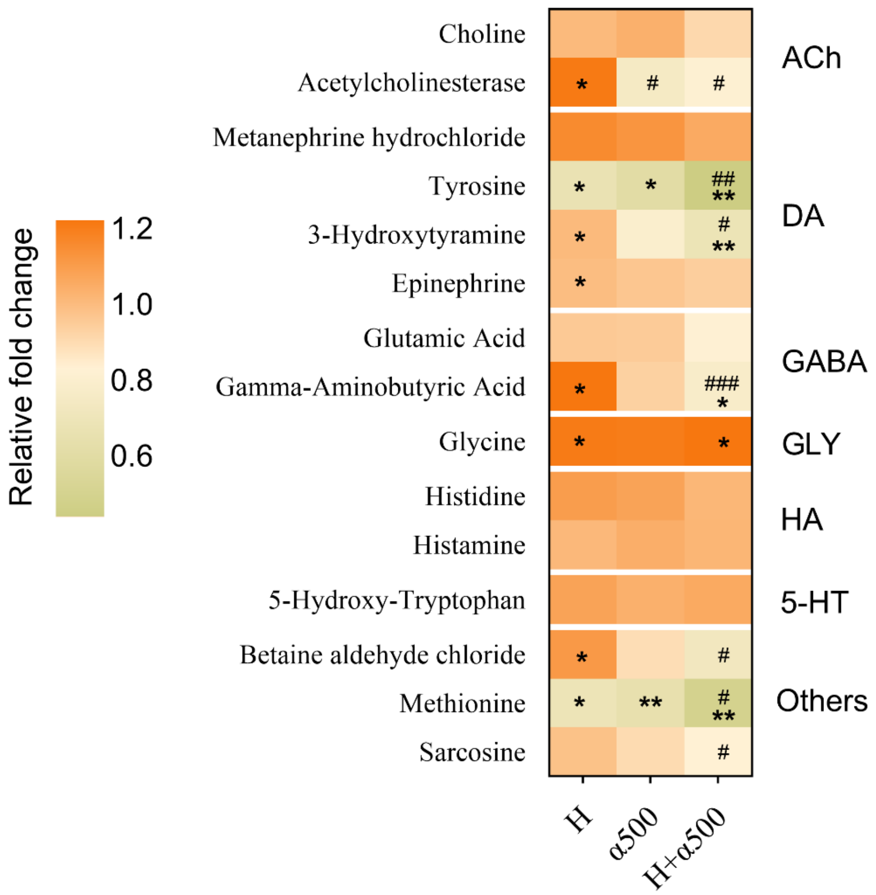

Figure S10. Heatmap of acetylcholinergic (ACh), dopaminergic (DA), glutaminergic and GABAergic (GABA), glycinergic (GLY), histaminergic (HA), and serotoninergic (5-HT) exposure to chlorpyrifos, as well as other-energetic neurotransmitters in zebrafish larvae. H indicates 100 μg/L chlorpyrifos, α500 indicates 500 μg/L GW6471, H+α500 indicates coexposure 500 μg/L GW6471 with 100 μg/L chlorpyrifos. The error bars are presented as the SEM. Significant differences to VC are indicated with $*p < 0.05$, $**p < 0.01$ and $***p < 0.001$. Significant differences to H are indicated with $^{\#}p < 0.05$ and $^{\#\#\#}p < 0.001$.

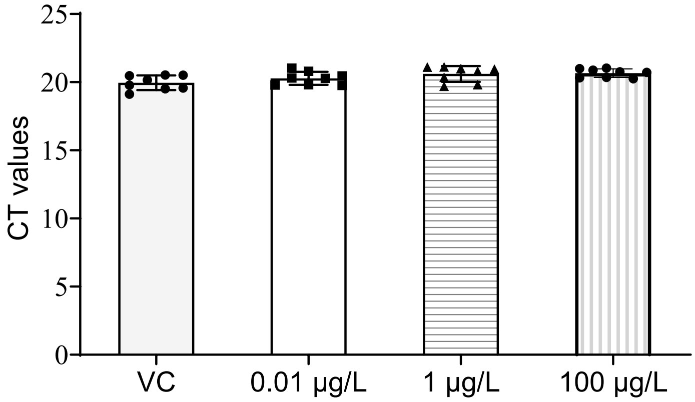

Figure S11. The CT values of β-actin in zebrafish (Danio rerio) exposed to 0.01, 1, and 100 μg/L chlorpyrifos.
